# Cobalt modulates methanol turnover of the alcohol dehydrogenase in *Desulfofundulus kuznetsovii* strain TPOSR

**DOI:** 10.1128/aem.00215-25

**Published:** 2025-04-09

**Authors:** Lukas Friedeheim, Karel Olavarria, Alfons J. M. Stams, Diana Z. Sousa

**Affiliations:** 1 Laboratory of Microbiology, Wageningen University & Research 568404, Wageningen, the Netherlands; 2 Centre for Living Technologies, Alliance EWUU, Utrecht, the Netherlands; University of Nebraska-Lincoln, Lincoln, Nebraska, USA

**Keywords:** methanol metabolism, alcohol dehydrogenase, cobalt, sulfate-reducing bacteria

## Abstract

*Desulfofundulus kuznetsovii* strain 17^T^ oxidizes methanol via a two-pathway system involving both alcohol dehydrogenases (ADH) and a cobalt-dependent methanol methyltransferase (MT). In contrast, *D. kuznetsovii* strain TPOSR lacks the MT pathway, relying solely on ADH for growth on methanol. Despite the absence of the MT pathway, cobalt starvation resulted in lower methanol uptake rates and reduced growth rates in strain TPOSR, suggesting a critical role of cobalt in methanol metabolism outside of its role in the MT system. Given the often-crucial role of metal cofactors such as iron, zinc, and other metals in the active site of ADHs, we hypothesized that cobalt could influence the catalytic activity of the TPOSR ADHs. The gene encoding for the most abundant ADH during growth on methanol, Adh1, was heterologously expressed in *Escherichia coli*, and the enzyme was purified for kinetic studies. Adh1 exhibited optimal activity at 55°C and is oxygen tolerant. The methanol turnover rate increased from 1.76 (95% Cl [1.56, 1.99]) s⁻¹ to 3.5 (95% Cl [3.3, 3.72]) s⁻¹ with the addition of 2 µM CoSO_4_, while higher cobalt concentrations (>5 µM) inhibited Adh1 activity. Similarly, NiSO_4_ addition (1–1000 µM) enhanced Adh1 activity, with a 75% improvement observed at an optimum concentration of 200 µM. Our findings suggest that the importance of cobalt for the methanol metabolism of sulfate-reducing organisms extends beyond its involvement in the MT system.

**IMPORTANCE:**

Methanol is a ubiquitous compound in natural environments, where it is produced geothermally or from plant and microbial biomass. Its microbial metabolism is particularly important in low-nutrient, oxygen-free environments, such as the deep subsurface, where specialized microbes compete for methanol and play a crucial role in the global carbon cycle. Typically, microbes in these settings rely on a cobalt-dependent methanol methyltransferase (MT) pathway for methanol breakdown. However, *Desulfofundulus kuznetsovii* TPOSR deviates from this, lacking the MT pathway and instead relying solely on alcohol dehydrogenases (ADH) to oxidize methanol. Despite the absence of the cobalt-dependent MT system, our study shows that cobalt strongly stimulates the activity of the most abundant ADH, revealing an unexpected, yet significant role for cobalt in this alternative methanol metabolism. Understanding these interactions not only sheds new light on methanol metabolism in nature but also opens up possibilities for developing more efficient and sustainable technologies for methanol conversion in industry.

## INTRODUCTION

In recent years, there has been a growing interest in the utilization of C1 compounds such as carbon dioxide, carbon monoxide, formic acid, methanol, and methane as feedstocks for biotechnological applications (1, 2). Among these compounds, methanol stands out due to its low cost and ease of handling. Methanol can be efficiently produced from water and CO₂ through electrochemical processes. It is easy to transport, and it is highly soluble in water (3, 4). Many microorganisms tolerate high methanol concentrations, making it a suitable microbial substrate, both in oxic and anoxic environments. In aerobic and facultative anaerobic organisms, methanol assimilation is mediated by alcohol dehydrogenases (ADH), while strict anaerobic microbes, like acetogens and methanogens, can also employ cobalt-dependent methanol methyltransferases (MT). However, it was demonstrated that the sulfate reducer *Desulfofundulus kuznetsovii* strain 17^T^ has genes encoding for both the ADH and the MT pathway (5). Proteome analysis showed that proteins of both pathways are highly abundant during growth on methanol in the presence of cobalt, while only the ADH was abundant during growth without cobalt. In contrast, a newly described strain, *D. kuznetsovii* TPOSR, lacks the essential genes encoding for MT, yet it was able to grow on methanol. Proteomics analysis of methanol-grown cells of *D. kuznetsovii* TPOSR revealed an abundant ADH with high amino acid similarity to the ADH from strain 17^T^ (6). Furthermore, physiological experiments growing strain TPOSR with methanol indicated impaired growth and methanol uptake when cobalt was omitted. Assuming that the ADH was a cobalt-independent enzyme, these impairments were attributed to the effect of the lack of cobalt on other cobalt-dependent enzymes such as methionine synthase (7). However, ADHs are metalloproteins, and a modulation of their activity by cobalt was previously proposed (8), leaving a direct effect of cobalt availability on the activity of the ADH as a possible explanation for the observed growth impairment of strain TPOSR with methanol in the absence of cobalt.

ADHs catalyze the reversible conversion between alcohols and aldehydes or ketones. These reactions require the transfer of electrons between the substrate and specific cofactors. Substrate and cofactor are coordinated within the active site of the enzyme by a metal ion. Since the first purification of an ADH from yeast (9), a plethora of ADHs has been studied and divided into three groups based on the cofactor specificity: (i) NAD(P)-dependent; (ii) PQQ, F_420_, and haem-dependent; and (iii) FAD-dependent. NAD(P)-dependent ADHs are further divided into three types: (i) medium-chain or zinc-dependent as present in horse liver (10), (ii) short-chain as found in *Drosophila melanogaster* (11), and (iii) iron-containing/iron-activated ADHs as first reported from *Zymomonas mobilis* (12). ADHs of *Desulfofundulus kuznetsovii* belong to the latter group. The current understanding of type III ADHs and their potential applications was reviewed recently in reference 13, including the current knowledge about methanol metabolism facilitated by ADH systems. Despite the name “iron-containing/iron-activated,” there are representatives of type III ADHs that contain zinc, cobalt, or no metal ion at all (14, 15). The crystal structures of ADHs display a conserved tunnel-like structure with the coordinating metal at its center and the substrates entering from opposing sites of this tunnel. The kinetic activity is strongly dependent on the type of metal available and varies between different ADH isoforms, as does the substrate specificity. Oxidation of methanol exhibits significantly lower conversion rates when compared to many other alcohols, with some methanol dehydrogenases being dependent on activating proteins (ACT, NUDIX hydrolase) to perform their catalytic activity, as found in *Bacillus methanolicus* (16).

ADHs have potential for application in bioremediation or to produce alcohols and other related fine chemicals (17), but their appeal is often limited by their low thermal stability, low turnover rates, and oxygen intolerance (18). Consequently, there is an increasing interest in ADHs from thermophilic and hyperthermophilic organisms because of their higher turnover rates and greater thermal stability. The exploration of the enzymes involved in the methanol oxidation in the strain *D. kuznetsovii* TPOSR offers the possibility of characterizing a thermophilic ADH and provides an opportunity to gain deeper insights into the mechanisms behind cobalt dependency in methanol metabolism. Understanding whether the observed growth inhibition during cobalt starvation is due to the inhibition of the ADH itself or other cellular processes contributes to this effort.

In this study, we describe the effect of cobalt on the growth and methanol uptake rates of *D. kuznetsovii* TPOSR, along with akinetic analysis of its Adh1 protein. In the presence of cobalt, methanol is completely consumed, whereas the absence of cobalt in the culture medium inhibits methanol consumption, halting the strain’s ability to grow at low methanol concentrations. Studies with the purified Adh1 showed reduced activity in the absence of cobalt, consistent with the previously mentioned physiological observations. On the other hand, given its oxygen and thermal tolerance, as well as its methanol oxidation rates, this enzyme could be a promising candidate for biotechnological applications.

## MATERIALS AND METHODS

### Microorganisms and cultivation

*Desulfofundulus kuznetsovii* strain TPOSR (DSM 110707) was available at our culture at the Laboratory of Microbiology, Wageningen University & Research, The Netherlands. The strain was grown in serum bottles that were half-filled with sterile basal anoxic bicarbonate-buffered medium (19), with a headspace filled with N_2_/CO_2_ (80/20% [v/v], 1.7 atm). Vitamin solution, prepared as previously described by (19), along with methanol (5 mM) and sulfate (20 mM) were added to the medium from sterile anoxic stock solutions. Cobalt and vitamin B12-containing medium included 0.5 µM CoCl_2_ and 100 µg L^−1^ cyanocobalamin unless stated otherwise. Cobalt-free medium was obtained by omitting cobalt and vitamin B12 (cyanocobalamin) from the trace element and vitamin solutions. References in this manuscript to “cobalt-free” always mean free of both cobalt and vitamin B12. Bottles were cleaned with an acidic solution (aqua regia—HNO_3_ and HCl 1/3 mol/mol) prior to use, to remove any trace cobalt and other residues. Bottles were inoculated with 1% (v/v) inoculum from exponentially growing cultures of strain TPOSR. Cultures were incubated statically at 55°C in dark conditions. All used chemicals had analytical grade and were purchased from Sigma-Aldrich (St. Louis, MO).

### Analytical techniques

Microbial growth was tracked by measuring the optical density at 600 nm in a UV-1800 UV–Vis spectrophotometer (Shimadzu, Japan). Methanol concentration was measured using a GC-2010 (Shimadzu, Japan) equipped with a DB-WAX UI column (Agilent, Santa Clara, CA) with N_2_ as carrier gas at a constant pressure of 100 kPa and using a flame ionization detector (FID) at a temperature of 250°C. Liquid samples of the culture medium were injected using a headspace autosampler HS-20 (Shimadzu, Japan) with an oven set to 50°C to evaporate the methanol for the analysis.

### ADH phylogenetic analysis

For the analysis of the phylogenetic relationship of TPOSR ADHs with ADHs of related species and ADHs with reported ability to oxidize methanol, we constructed an amino acid-based neighbor joining tree. The percentage of replicate trees in which the associated taxa clustered together in Felsenstein’s bootstrap test (20) (1000 replicates) is indicated on the branches between 0.5 and 1. The tree is drawn to scale, with branch lengths in the same units as those of the evolutionary distances used to infer the phylogenetic tree. The evolutionary distances were computed using the Poisson correction method and are in the units of the number of amino acid substitutions per site. The analysis involved 74 amino acid sequences. All positions containing gaps and missing data were eliminated. There was a total of 373 positions in the final data set. Evolutionary analyses were conducted in MEGA11 (21).

### Plasmid construction

The plasmid used for TPOSR Adh1 over-expression was based on the pET26b backbone derived from reference 22 and obtained from reference 23. The amino acid encoding DNA sequence of TPOSR Adh1 (G7K71_RS16190) was obtained from the National Center for Biotechnology Information and harmonized for the expression in *E. coli* BL21 using the Codon Harmonizer tool (24). The DNA sequence was subsequently modified to contain an N-terminal TEV protease target sequence followed by a six-histidine tail. A synthetic gBlock containing this DNA sequence was obtained from Integrated DNA Technologies. The synthetic sequence was then assembled into the pET26b backbone using NEBuilder HiFi DNA assembly and introduced into *E. coli* BL21 according to standard protocol. The harmonized sequence containing the TEV cleavage site and the HIS-tag is available in Supplement S1.

### Protein expression and purification

Alcohol dehydrogenase was purified using a previously described protocol (25) with some modifications. *E. coli* BL21 cells carrying the previously described expression plasmid were aerobically cultivated in 1 L of Lysogenic Broth (LB) medium supplemented with 50 mg L^−1^ kanamycin, at 37°C, with shaking at 120 rpm. The medium was not further supplemented with any metal ions or other additives. Upon reaching an OD_600_ between 0.6 and 0.8, cultures were rapidly cooled in ice-cold water for 15 minutes. Subsequently, protein expression was induced by adding isopropyl-β-D-thiogalactopyranosid (IPTG) to a final concentration of 0.2 mM, followed by overnight incubation at 19°C with shaking at 120 rpm. After incubation, cells were harvested by centrifugation at 6,000 *× g,* at 4°C, for 15 min. The resulting pellet was washed once with 50 mM HEPES buffer (pH 7.4) and centrifuged again at 6,000 *× g,* at 4°C, for 15 min. The washed pellet was then stored at −70°C until further use.

For cell lysis, the frozen cell pellet was thawed and resuspended in 20 mL of binding buffer (50 mM HEPES pH 7.4, 300 mM NaCl, 20 mM imidazole) containing one tablet of cOmplete protease inhibitor. Cells were sonicated on ice using a Qsonica Sonicator Q500 with a half-inch probe at 25% amplitude for 10 min (1 s ON/ 2 s OFF). The cell lysate was centrifuged at 30000 *× g,* at 4°C, for 45 min. The resulting supernatant was filtered through a 0.22 µm membrane filter and used for protein purification.

The protein was purified by Fast Performance Liquid Chromatography (FPLC), using an ÄKTA Go Protein Purification system (GE Healthcare Life Sciences). The cell-free extract was loaded on a 5 mL His Trap HP column (GE Healthcare Life Sciences) that was pre-equilibrated with binding buffer. The protein was then eluted by washing the column with elution buffer (50 mM HEPES, pH 7.4, 300 mM NaCl, 500 mM imidazole). The collected protein was desalted in batches of 1.5 mL using a 5 mL HiTrap desalting column (Cytiva) pre-equilibrated with 50 mM HEPES pH 7.4. The protein was then diluted to be used in enzymatic assays or stored undiluted in 50 mM HEPES pH 7.4 at 4°C.

Protein concentrations were determined using the Pierce BCA Protein Assay Kit (Thermo Fisher Scientific), with bovine gamma globulin as standard. The purity of the resulting protein was checked using SDS-PAGE. Protein samples were denatured in Laemmli Sample Buffer (Bio-Rad) at 98°C for 5 min. The sample was centrifuged at 10,000 × g, at room temperature, for 30 s and separated on Mini-PROTEAN TGX precast gel (Bio-Rad), at 20 mA for 45 min, and stained using Page Blue protein staining solution (Thermo Fisher Scientific), resulting in a single band in a position correlated to the expected molecular weight of a monomer of Adh1 (42.8 kDa).

### Enzymatic assays

To determine the enzymatic activity of the heterologously expressed Adh1 from the strain TPOSR, the changes in the concentration of NADH were measured at a wavelength of 340 nm using a Synergy MX Multi-Mode Microplate Reader (BioTek). The optimum temperature was determined using NAD^+^ (0.5 mM and 1 mM) and methanol (1 mM, 10 mM, and 100 mM) in 50 mM Bis-tris propane buffer, pH 9.4, across a temperature range between 30°C and 70°C. The pH optimum for the assessed enzymes was determined using NAD^+^ (1 mM) coupled to the oxidation of methanol (1 mM, 10 mM, and 100 mM) at 55°C in TRIS-HCl (pH 7.5, 8, 8.5, and 9), BIS-Tris propane (BTP) (8.6, 9, 9.4, and 10) and CAPS (9.5, 10, 10.5, and 11). We checked whether the HIS-tag extension affected methanol oxidation by comparing uncleaved Adh1 with TEV-cleaved Adh1 (overnight incubation at 4°C) with methanol (0–4.9 M) and NAD^+^ (2 mM) in BTP (pH 9.4) at 55°C. Since no significant difference was observed (Supplement S3), the uncleaved Adh1 was used. Kinetic assays to study the methanol oxidation of TPOSR Adh1 were carried out in 50 mM BTP buffer, at 55°C, using 96-well microplates (Greiner, 675101). The concentration of Adh1 in the assays was 50 or 75 nM (2.14 or 3.21 µg/µl) unless stated otherwise. Substrate stocks were freshly prepared and kept on ice, and NAD^+^ and NADH concentrations were quantified spectrophotometrically. For the determination of the kinetic parameters, we used NAD^+^ concentrations ranging from 0.02 to 2.5 mM and methanol concentrations from 0.75 to 3000 mM. To evaluate the effect of metal ions on the rate of methanol oxidation catalyzed by TPOSR Adh1, initial rates of methanol (100 mM) oxidations with NAD^+^ (0.8 mM) were determined with the addition of one of the following metals in a concentration range from 1 µM to 1 mM: CoSO_4_, Fe(II)SO_4_, Fe(III)Cl_3_, ZnCl_2_, MnSO_4_, CuSO_4_, and NiSO_4_. We further tested the addition of EDTA (1 µM to 1 mM) without metal supplementation to assess the impact of chelation on the purified enzyme. Relative activities of the reactions in the presence of all metals or EDTA are available in the supplemental material (Supplement S3). Reactions in the presence of cobalt were carried out, unless otherwise stated, with a final CoSO_4_ concentration of 2 µM. Inhibition of TPOSR Adh1 by NADH was assessed by combining a constant methanol concentration (1 M) with NAD^+^ at different concentrations (0–1.5 mM), at increasing concentrations of NADH (0–500 µM). The kinetic parameters of the purified enzyme were determined using the software Dynafit (26). Model discrimination analyses were performed to determine which enzymatic mechanism best explains the experimental observations. The Dynafit scripts used for these analyses as well as the measured methanol oxidation rates are available in the supplemental material (Supplement S2).

### Cell-free extracts

For the preparation of cell-free extracts, cells of strain TPOSR were grown as described above with methanol (40 mM) and sulfate (20 mM) in 50 mL anaerobic serum bottles. The cultures were harvested by centrifugation at 10,000 × *g* for 15 minutes at 4°C. The resulting pellet was resuspended in 2 mL of BTP buffer (pH 9.4) containing cOmplete protease inhibitor (1 tablet per 20 mL) and sonicated while on ice using a Qsonica Sonicator Q500 with an MS72 micro tip probe at 25% amplitude for 10 min (1 s ON/ 2 s OFF). The resulting extract was centrifuged at 30,000 × *g* for 5 min at 4°C, filtered through a 0.45 µm membrane filter, and used for assays immediately. Assays were conducted as described for the purified Adh1 at 55°C in BTP buffer (50 mM, pH 9.4) using NAD^+^ concentrations ranging from 0.02 to 2 mM and methanol concentrations from 0.75 to 3000 mM.

## RESULTS

### Cobalt starvation inhibits growth and methanol uptake rate in *D. kuznestovii* strain TPOSR

**Fig 1:**
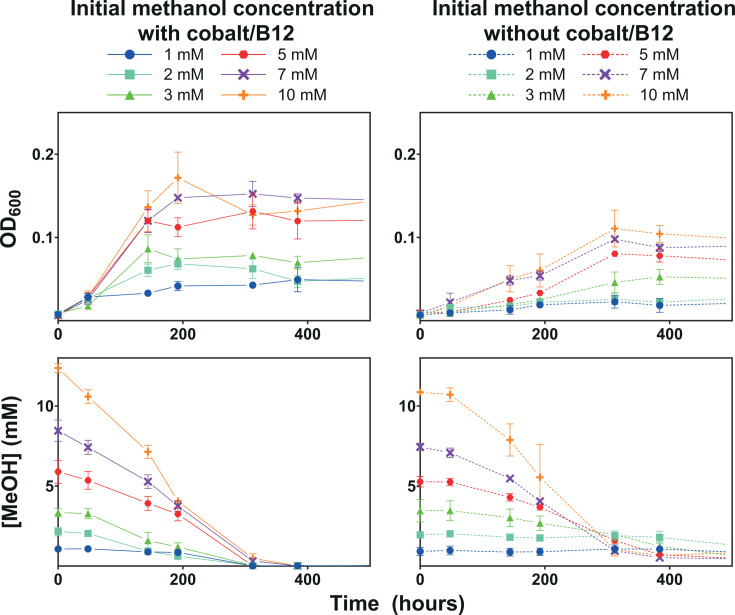
Growth of strain TPOSR (A + B) and methanol concentration in the medium (C + D) with different initial methanol concentrations (1 mM—blue circle, 2 mM—turquoise square, 3 mM—green triangle, 5 mM—red hexagon, 7 mM—purple X and 10 mM—orange plus) in presence (A + C) and absence (B + D) of cobalt and vitamin B12. Optical density and methanol concentration are from the same set of bottles (A + C and B + D) with corresponding colors. Data points represent the mean of triplicate with standard deviation.

The metabolism of methanol in *D. kuznetsovii* strain TPOSR, a sulfate-reducer isolated from a low cobalt environment, has been recently described (6). The absence of the genes encoding for a complete cobalt-dependent MT system in the genome of this strain, and the high abundance of an ADH annotated as type III (iron-containing) in methanol-grown cells, led to the hypothesis that strain TPOSR would be adapted to grow in low cobalt conditions. However, the growth of strain TPOSR was impaired in the absence of cobalt. To further explore how cobalt starvation affects strain TPOSR, we observed the growth and the methanol uptake of strain TPOSR, in the presence or the absence of cobalt and vitamin B12, with varying initial methanol concentrations (Fig. 1).

In the presence of cobalt, methanol concentration decreased to a value below the detection level (~0.05 mM), with optical density increasing accordingly. Maximum density was achieved within 150–200 hours with complete methanol consumption. Cobalt and vitamin B12 starvation had a clear inhibitory effect on growth and methanol consumption. In this condition, no growth or methanol consumption was detected in cultures starting with 1 or 2 mM of methanol. Cultures with initial methanol concentrations of 3, 5, 7, and 10 mM achieved a 30% lower final optical density. Additionally, at the tested methanol concentrations, maximum density was reached only after 300–400 hours, due to a severe reduction in their growth rates. Interestingly, during cobalt and B12 starvation, methanol consumption was never complete and halted when methanol concentrations reached 0.3–0.5 mM, independent of the initial methanol concentration (Fig. 1). As the initial enzymatic step of methanol metabolism, a reduction of the ADH activity during cobalt starvation could lead to lower carbon assimilation and lower energy generation, reflected in the observed phenotype. This reasoning led us to a more detailed study of the ADH activity in *D. kuznetsovii* TPOSR.

### Functional characterization of the methanol oxidizing TPOSR Adh1

**Fig 2:**
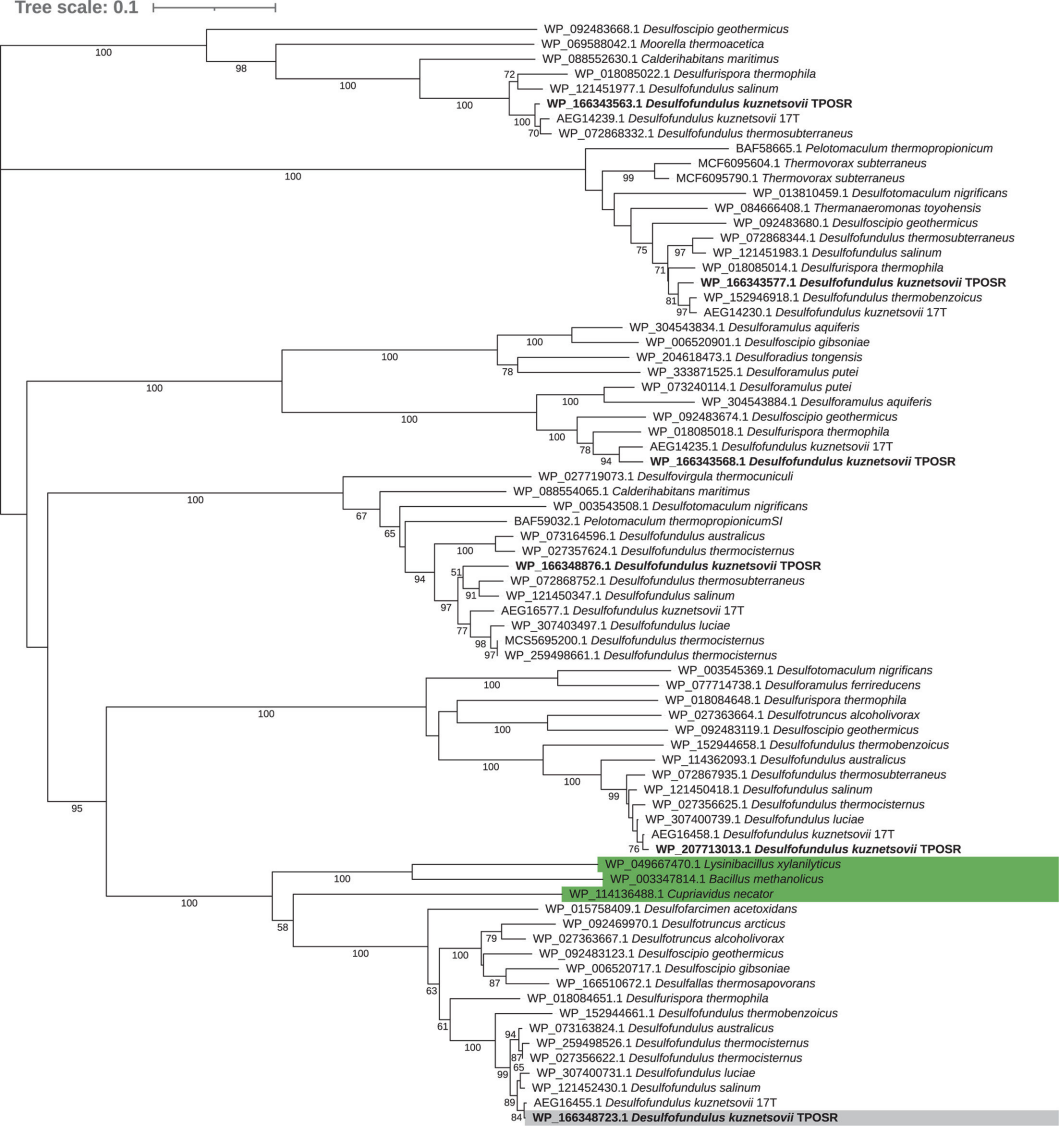
Neighbor joining tree showing the phylogenetic relationship of TPOSR ADHs (bold, Adh1 with grey background) with closest relatives and selected ADHs with documented activity with methanol (highlighted in green). Bootstrap support is indicated for values above 50%.

To evaluate whether the observed effects of cobalt and vitamin B12 on growth and methanol uptake in *D. kuznetsovii* TPOSR are due to specific inhibition of one of its ADHs, we first focused on identifying which could be the key ADH responsible for methanol oxidation, as *D. kuznetsovii* TPOSR encodes six ADHs all annotated as iron-containing ADHs according to blastp (27). Upon phylogenetic analysis, ADHs were placed into separate clusters (Fig. 2). While the genome of strain TPOSR contains six genes encoding ADHs, only one, WP_166348723.1 referred to as Adh1, has been previously shown to be highly abundant during growth with methanol. The abundance of other ADHs did not increase when the strain was grown with methanol (6). Furthermore, the gene encoding for Adh1 also clusters with iron-containing ADHs which oxidize methanol from *Cupriavidus necator*, *Bacillus methanolicus,* and *Lysinibacillus xylanilyticus* (16, 28, 29). These proteome and taxonomic indications strongly suggest that Adh1 plays a primary role in methanol oxidation in strain TPOSR. Therefore, we decided to isolate this enzyme and study its kinetic properties.

**Fig 3:**
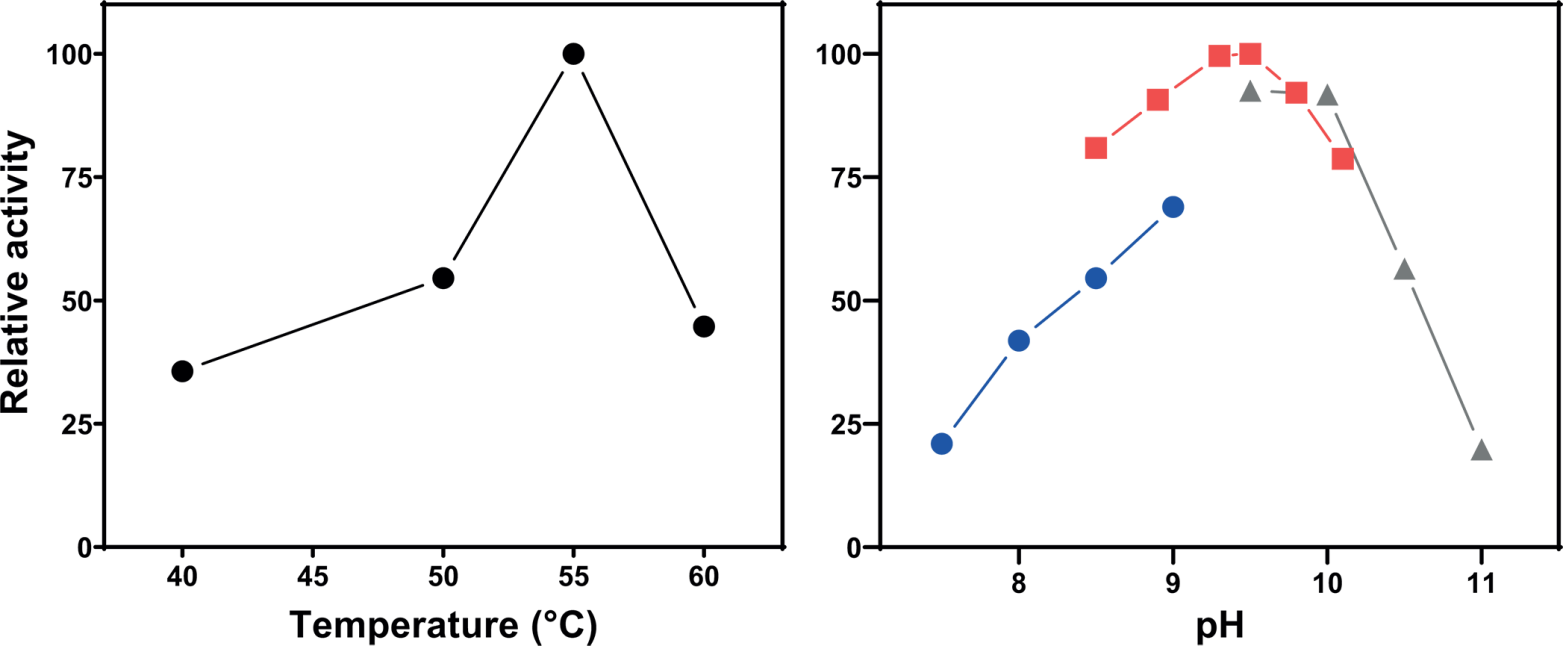
Effect of reaction temperature and reaction pH (TRIS-HCl—blue circles, BTP—red squares, CAPS—grey triangles) on methanol oxidation rate catalyzed by TPOSR Adh1. Data reflect relative activity (%) respective to the highest measured rate set to 100%.

TPOSR Adh1 (WP_166348723.1, 387 amino acids) was expressed in *E. coli* BL21. The expressed protein contained a C-terminally bound 6-histidine tag connected via a TEV cleavage site. No catalytic differences were observed in methanol oxidation assays between Adh1 with the TEV-histidine extension and Adh1 with the extensions cleaved off, and therefore the non-cleaved enzyme was used in our further study. The optimal conditions for the oxidation of methanol by the purified TPOSR Adh1 were a temperature of 55°C and a pH of 9.3–9.5 (Fig. 3).

The structure of Adh1, as predicted by alphafold2 (30), reflects the typical features of an iron-containing ADH, with a deep cleft in the center of the protein. Centrally located in the cleft is the active site composed of three histidine residues and one asparagine residue that are highly conserved and responsible for the coordination of the metal ion.

### Low concentrations of cobalt and nickel promote methanol oxidation

**Fig 4:**
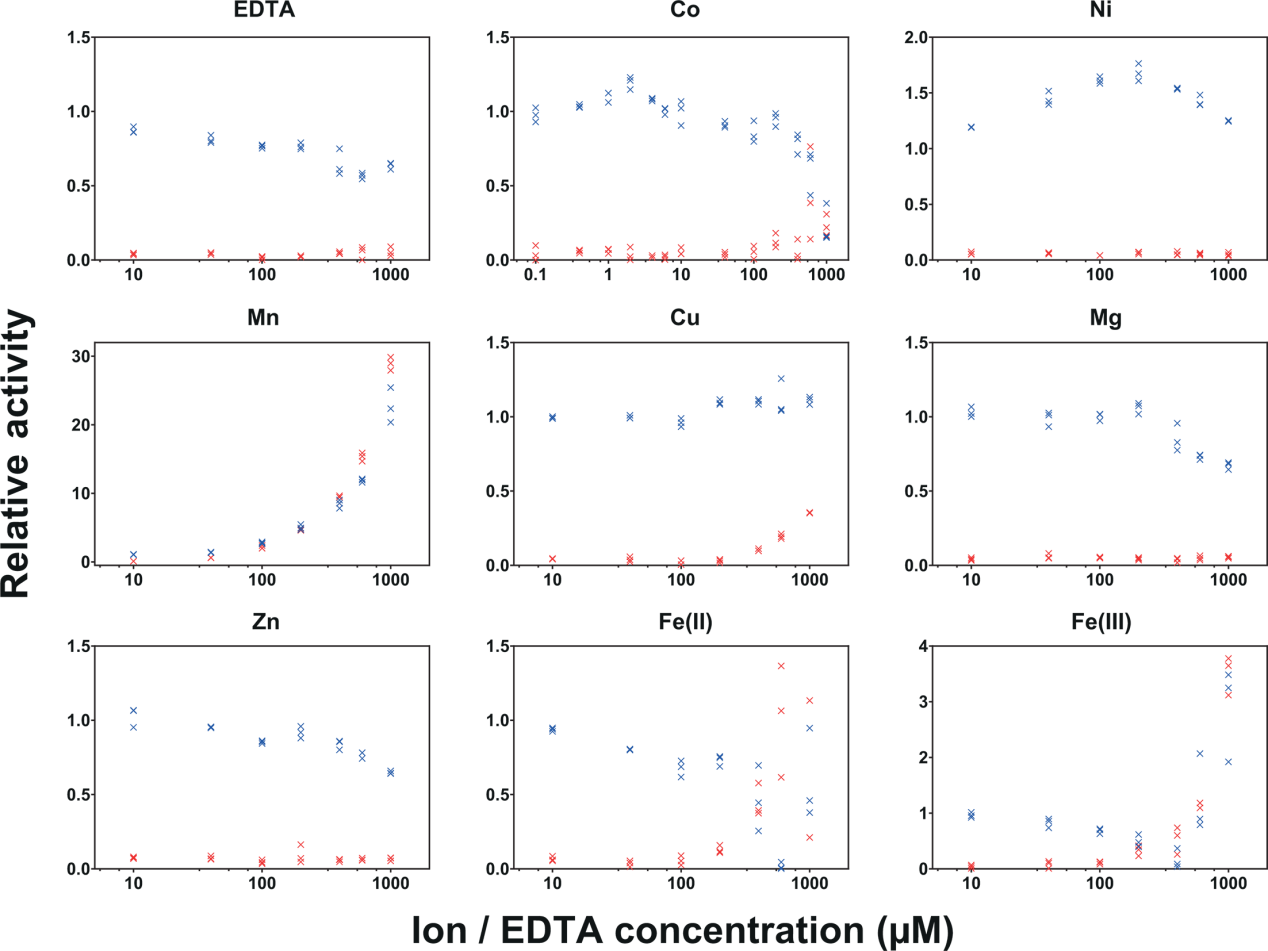
Relative initial rates of methanol oxidation with increasing concentrations of metal ions at identical concentrations of enzyme and substrates (blue) as obtained by product formation measured at abs_340_. The effect of metal ion concentration on absorption at 340 nm without the addition of enzyme is included as a control (red). Data points reflect relative activity normalized to the activity measured without the addition of any ions or EDTA set to 100% indicated as 1.

It is shown frequently that ADH activity is strongly dependent on the type and oxidation state of the associated metal ion which is essential for cofactor binding (28, 29, 31). In agreement with these observations, the activity of TPOSR Adh1 was strongly dependent on its metal core. This was reflected in the reduction of methanol oxidation activity after the treatment with metal-chelating ethylenediaminetetraacetic acid (EDTA). To assess the effect of different metal cofactors, we determined the methanol oxidation rates at various metal ion concentrations (Fig. 4). While the annotation “iron-containing ADH” suggests a Fe(II) or Fe(III) core, the addition of either of these ions did not result in a beneficial effect on the methanol oxidation rate of Adh1. By contrast, the addition of either Fe(II)SO_4_ or Fe(III)Cl_3_ led to reduced methanol oxidation rate already at low concentrations, while higher concentrations led to photometric artifacts caused by the turbidity of the mixture. Similarly, the addition of ZnCl_2_ or MgSO_4_ inhibited methanol oxidation of Adh1 above 200 or 300 µM, respectively. No change in activity was observed with the addition of CuSO_4_ in the range of 1–500 µM. On the other hand, the addition of cobalt and nickel at low concentrations showed beneficial effects on the rate of methanol oxidation by Adh1. The addition of CoSO_4_ led to a 25% increase in methanol oxidation rate at an ion concentration of 2 µM, whereas higher ion concentrations resulted in inhibition. Similarly, the addition of nickel achieved a 70% increase in activity at a concentration of 200 µM (2000 times enzyme concentration), while higher concentrations were inhibiting. A strong increase in absorption at 340 nm was observed during the addition of increasing concentrations of MnSO_4_. However, a similar signal was observed in enzyme-free controls, and the increase in signal was therefore attributed to the metal itself. These results indicate that cobalt and nickel, at low concentrations, can enhance the activity of the purified TPOSR Adh1. Unexpectedly, while the heterologously expressed ADH retained activity for weeks when stored in oxic buffer at 4°C, the cell-free extracts from *D. kuznetsovii* TPOSR lost alcohol oxidation activity within minutes after the extraction (data not shown).

### Cobalt elevates methanol turnover numbers of TPOSR Adh1

Motivated by the activating effect of cobalt on the methanol oxidation rate of TPOSR Adh1, we explored the effect of cobalt on the kinetics of methanol oxidation using NAD. No activity with NADP^+^ (10, 100, and 1000 µM) was detected using methanol at 100 and 1000 mM concentrations. To determine the effect of cobalt addition, we studied the activity of Adh1 with or without the addition of 2 µM cobalt, the concentration at which we observed the highest increase in activity before (Table 1). Model discrimination analysis indicated that the kinetic behavior of the enzyme was best explained by a rapid-equilibrium mechanism in which NAD^+^ binds first. The data indicate that the *k_cat_* of the purified Adh1 doubled in the presence of 2 µM cobalt. The *K_M_* for methanol increased while the *K_M_* for NAD^+^ decreased but remained within their respective confidence intervals.

**TABLE 1:** Kinetic parameters of TPOSR Adh1 with and without the addition of cobalt^*a*^

	Without Co	With 2 µM Co
*k_cat_* (s^−1^)	1.76 [1.56, 1.99]	3.5 [3.3, 3.72]
*K_M_* methanol (mM)	209 [113, 351]	333 [270, 408]
*K_M_* NAD^+^ (mM)	0.550 [0.26, 1.3]	0.142 [0.086, 0.223]
*k_cat_* /*K_M_* methanol (M^−1^ s^−1^)	8.42	10.51

^*a*^ Reported values include confidence intervals at a 95% probability level.

While the absence of cobalt reduces the rate of methanol oxidation catalyzed by Adh1, another factor that can contribute to the observed growth impairment is product inhibition by NADH. We determined methanol oxidation rates at saturating concentrations of methanol and varying concentrations of NAD^+^ and NADH. Our results indicate a clear inhibitory effect of NADH. Global fitting of the measured data indicates a mixed inhibition pattern, with *K_iC_* = 11.1 [4.87, 26.3] and *K_iU_* = 77 [53, 120], suggesting the presence of another binding site for NADH different from the active site, with potential regulatory effects.

## DISCUSSION

The physiological analysis of *D. kuznetsovii* TPOSR revealed impaired methanol metabolism and the inability to metabolize low methanol concentrations during cobalt starvation. Due to the absence of the cobalt-dependent MT system in strain TPOSR, it had been initially thought that cobalt would not play a significant role in methanol oxidation. However, similar impairments while omitting cobalt have been previously attributed to reduced activity of other cobalt-dependent enzymes, but a direct effect of cobalt on the ADH system remains possible. The phylogenetic grouping of the TPOSR ADHs in separate branches could indicate different substrate preferences, but the lack of enzymatic data precludes further hypotheses. The clustering of Adh1 with the methanol-oxidizing ADHs of *Cupriavidus necator*, *Bacillus methanolicus*, and *Lysinibacillus xylanilyticus*, combined with its high abundance in the proteome of methanol-grown cells of TPOSR (6), points out the importance of Adh1 as potentially the primary ADH involved in methanol oxidation.

The purified TPOSR Adh1 displayed optimal methanol oxidation at elevated temperatures consistent with the thermophilic nature of strain TPOSR, and at elevated pH values, which aligns with other type III ADHs (12, 31, 32). Kinetic studies revealed that the methanol turnover of TPOSR Adh1 is at the higher end of the reported *k_cat_* ranges (0.013 [33]–3.1 [34]) for NAD/NADP dependent alcohol dehydrogenases (EC 1.1.1.1) as well as for methanol specific ADHs (EC 1.1.1.244, 0.02 [35]–0.33 [29]) with similarly high methanol-specific *K_M_* values (0.78 [36]–294.23 [33]). While the determined *K_M_* is comparable to those reported for the highly similar ADH of strain 17^T^ (37), the *k_cat_* of TPOSR Adh1 surpasses the reported 0.045 s^−1^, which was determined at pH 7.4, although higher activity was observed at pH 9.

Methanol oxidation by Adh1 is strongly influenced by metal ion availability. Although this enzyme is annotated as an iron-containing ADH, the addition of iron (either Fe^2+^ or Fe^3+^) reduced methanol oxidation rates, while cobalt and nickel were found to increase Adh1 activity at low concentrations. The beneficial effects of Co^2+^ and Ni^2+^ on methanol oxidation align with observations made for the ADH from *Cupriavidus necator* (28), but the effect of different concentrations was not described. Reports detailing the effects of metal ions in a range instead of a single concentration are rare, even though they can drastically affect enzyme activity. Besides enzyme activity, cobalt could also influence the stability of Adh1. Reference 38 described a group-III ADH from *Pyrococcus horikoshii* OT3, NAD-dependent but not methanol-oxidizing, with low oxygen tolerance when coordinated with Fe^2+^. Oxygen exposure resulted in permanent inactivation of the iron-containing ADH, due to metal-catalysed oxidation of the enzyme, while exchange of the Fe^2+^ with Ni^2+^ and Co^2+^ reduced oxygen sensitivity. Tests with cell-free extracts of methanol-grown TPOSR show a loss of methanol oxidation activity within minutes, possibly indicating the use of Fe^2+^ in the native enzyme. The heterologously expressed form of TPOSR Adh1 retains activity for weeks when stored in oxic conditions, perhaps indicating incorporation of Co^2+^ or Ni^2+^ instead, both of which are likely available in the rich LB media that was used for cultivation of the *E. coli* expression strains. While this explains the stability differences between the native and the heterologous enzyme, the mechanism by which cobalt enhances its activity *in vivo* remains unclear. The binding of the ion to binding sites outside of the active site has been suggested as a general mechanism of protein–metal interaction (39). While this remains to be investigated, it could affect the stability of the protein or its substrate coordination. The effect of the exchange of iron and cobalt on the reaction mechanism of a 2-oxoglutarate oxygenase has been described by quantum chemical modeling (40), indicating that cobalt would reduce enzyme turnover due to higher energy requirements for the activation of oxygen. Although this is not directly relatable to methanol oxidation by an ADH, it can be imagined that the higher ionization energy (with Fe < Co < Ni) could affect the coordination of the substrates leading to the accordingly increased activity of the heterologous enzyme. Concluding, it is evident that additional research is required to further explore the effect of metal ions on the methanol oxidation of Adh1. A promising contribution would be to develop a setup that enables stable activity assays of the cell-free extract, allowing for a systematic assessment of the effects of cobalt and nickel addition on the native enzyme.

Product inhibition can be a contributing factor to the impaired growth of cells. We did not study the inhibition by formaldehyde because the much higher catalytic activities and lower saturation constants of aldehyde ferredoxin oxidoreductases (EC:1.2.7.5) in combination with its constitutively high abundance in the TPOSR proteome suggest that physiological formaldehyde concentrations are likely very low. Conversely, NADH is of particular interest because previous research using *Ralstonia eutropha* (now *Cupriavidus necator*) showed that an increased NADH/NAD^+^ ratio is a characteristic of growth driven by alcohol oxidation (41). While NADH is inhibiting at low concentrations, it does not explain the inhibited methanol uptake at low initial methanol concentrations, as no methanol oxidation has occurred yet, and NADH/NAD^+^ ratios should be low, resulting in minimal product inhibition of Adh1.

In conclusion, this study demonstrates the importance of cobalt for methanol oxidations of *D. kuznetsovii* TPOSR. While the exact mechanism remains unclear, our data suggest that the effect of cobalt on the methanol oxidation rate of Adh1 could contribute to the observed growth impairment of strain TPOSR. This does not exclude further effects of cobalt starvation on other cellular processes, which remain to be investigated. Furthermore, TPOSR Adh1 is positioned as an interesting enzyme for further research and biotechnological applications due to its high turnover of methanol and its low oxygen sensitivity. We emphasize the need for further research into the concentration-related effect of metal ions in enzymatic studies and propose *D. kuznetsovii* as an ideal model to study the metabolism of methanol in sulfate reducers due to its unique setup with the combined MT and ADH pathways in strain 17^T^ and the ADH dependence of strain TPOSR.

## SUPPLEMENTAL MATERIAL

Supplements S1 and S2 aem.00215-25-s0001.docx Harmonized sequence of TPOSR Adh1 with TEV cleavage site and HIS-tag S2, and Dynafit scripts for the calculation of methanol oxidation rates by Adh1.

Supplement S3 aem.00215-25-s0002.xlsx Data enzymatic assays.

ASM does not own the copyrights to Supplemental Material that may be linked to, or accessed through, an article. The authors have granted ASM a non-exclusive, world-wide license to publish the Supplemental Material files. Please contact the corresponding author directly for reuse.
