# Supplementary material for: Cobalt modulates methanol turnover of the alcohol dehydrogenase in Desulfofundulus kuznetsovii strain TPOSR
Source: Appl Environ Microbiol. 2025 Apr 9;91(5):e00215-25. doi: 10.1128/aem.00215-25 (PMC12093947; doi:10.1128/aem.00215-25)
Supplement: Supplements S1 and S2 — Harmonized sequence of TPOSR Adh1 with TEV cleavage site and HIS-tag S2, and Dynafit scripts for the calculation of methanol oxidation rates by Adh1. [file aem.00215-25-s0001.docx]

Cobalt modulates methanol turnover of the alcohol dehydrogenase in *Desulfofundulus kuznetsovii* strain TPOSR

Lukas Friedeheim^1^, Karel Olavarria^1,2^, Alfons J.M. Stams^1^, Diana Z. Sousa^1,2#^

^1^ Laboratory of Microbiology, Wageningen University & Research, Stippeneng 4, 6708WE, Wageningen, The Netherlands

^2^ Centre for Living Technologies, Alliance EWUU, Princetonlaan 6, 3584CB, Utrecht, The Netherlands

^#^corresponding author: [diana.sousa@wur.nl](mailto:diana.sousa@wur.nl)

# **Supplemental information**

## S1: Harmonized sequence of TPOSR Adh1 with TEV cleavage site and HIS-tag

## S2. Dynafit scripts for the calculation of methanol oxidation rates by Adh1

## **S1: Harmonized sequence of TPOSR Adh1 with TEV cleavage site and HIS-tag**

ATGGCTTTAGGGGAACAGGTATATGGTTACTATATTCCTACTGTGAACTTGATGGGGATTGGTGCTCATAAAAAAGTAGGCGAACAGGTGAAAATTTTGGGCGGCCGGCGGGCTTTGATTGTAACTGATGCGTATCTGGCGAAAAGCGGGATGGCGGATCAGATTAAAGCTCAGGTGGAAGAGGCGGGCGCGGAGGCGGTAATTTTTGCGGGTGCGGAGCCGAATCCGACCGATATTAACGTACACGATGGCCTTAAAGTGTTCCAGGAAAACCGCTGCGATATGATTATTAGCCTGGGCGGCGGCTCAAGCCATGATTGCGCGAAAGGGATTGGCATTGTGGCGACCAACGGTGGCAACATTCGCGATTACGAAGGCGTGGATAAAAGCAGCAAACCGATGCCGCCGTTCATTGCGGTGAACACCACCGCTGGTACTGCGAGCGAAATGACCCGGTTCTGCATTATTACCGATACAGATCGCAAAGTGAAAATGGCGATTGTGGATTGGCGCGTAACCCCGAACGTGGCGATAAACGATCCGCTGTTGATGGTGGGCATGCCTCCGGCGCTGACTGCTGCTACTGGTATGGATGCTCTTACCCATGCGGTGGAAGCTTACGTATCAACCATGGCGACCCCGGTAACCGATTCAGCTGCTCTGATGGCGATGAAACTTATTGCGGGCAACCTGCGTCAGGCGGTGGCGAACGGGCAGAACATGGAAGCGCGCGATCGGATGGCTTACGCGGAGTTCCTGGCGGGCATGGCTTTCAATAACGCGAGCCTGGGGTATGTACATGCGATGGCGCACCAATTAGGCGGCTTCTACAACTTGCCGCACGGCGTGTGCAACGCGATTCTGTTGCCGCACGTATGCCGGTTCAACCTGATTGCGTGCCCGCAGCGCTTTGCGGATATTGCTGTGGCTCTTGGCGAAAACATTGAGGGTTTGAGCGTGCGGGAGGCGGCGGAAAAAGCTATTTTCGCGATTCAGAAACTGTCAGAGGATGTGGGGATTCCTAGCGGGTTGGCGGGGCTGGGGGTGAAAGAGGAAGATCTGGCGATTATGGCGGAAAACGCGATGAAAGATGCGTGCAGCCTGACCAACCCGCGTCTGGCGACCCTGGATGATATAATTGATATTTATCGCCAGGCTATGAGCCAGTTTTATCTGAACGAACACCACCACCACCACCACTGA

## **S2. Dynafit scripts for the calculation of methanol oxidation rates by Adh1**

Methanol oxidation rates by Adh1 were determined for NAD^+^ concentration as stated in the file name. Each file contains methanol concentrations (first column) and measured rate (second column). Text files can be opened by double click once editing of the word document is enabled.

## Under cobalt-containing condition

##

## Under cobalt-free condition
